# Supplementary material for: Comprehensive Phenotypic Characterization of Diverse Drug-Type Cannabis Varieties from the Canadian Legal Market
Source: Plants (Basel). 2023 Nov 2;12(21):3756. doi: 10.3390/plants12213756 (PMC10648736; doi:10.3390/plants12213756)
Supplement: Supplementary file 1 [file plants-12-03756-s001.zip › supplementary_figures_Lapierre_et_al.pdf]

Comprehensive phenotypic characterization of diverse drug-type *Cannabis* varieties from the Canadian legal market

Éliana Lapierre, Maxime de Ronne, Rosemarie Boulanger, Davoud Torkamaneh *

Département de phytologie, Université Laval, Québec, Québec, Canada

Institut de Biologie Intégrative et des Systèmes (IBIS), Université Laval, Québec, Canada

Centre de recherche et d'innovation sur les végétaux (CRIV), Université Laval, Québec, Canada

Institut intelligence et données (IID), Université Laval, Québec, Canada

1. Supplementary figures

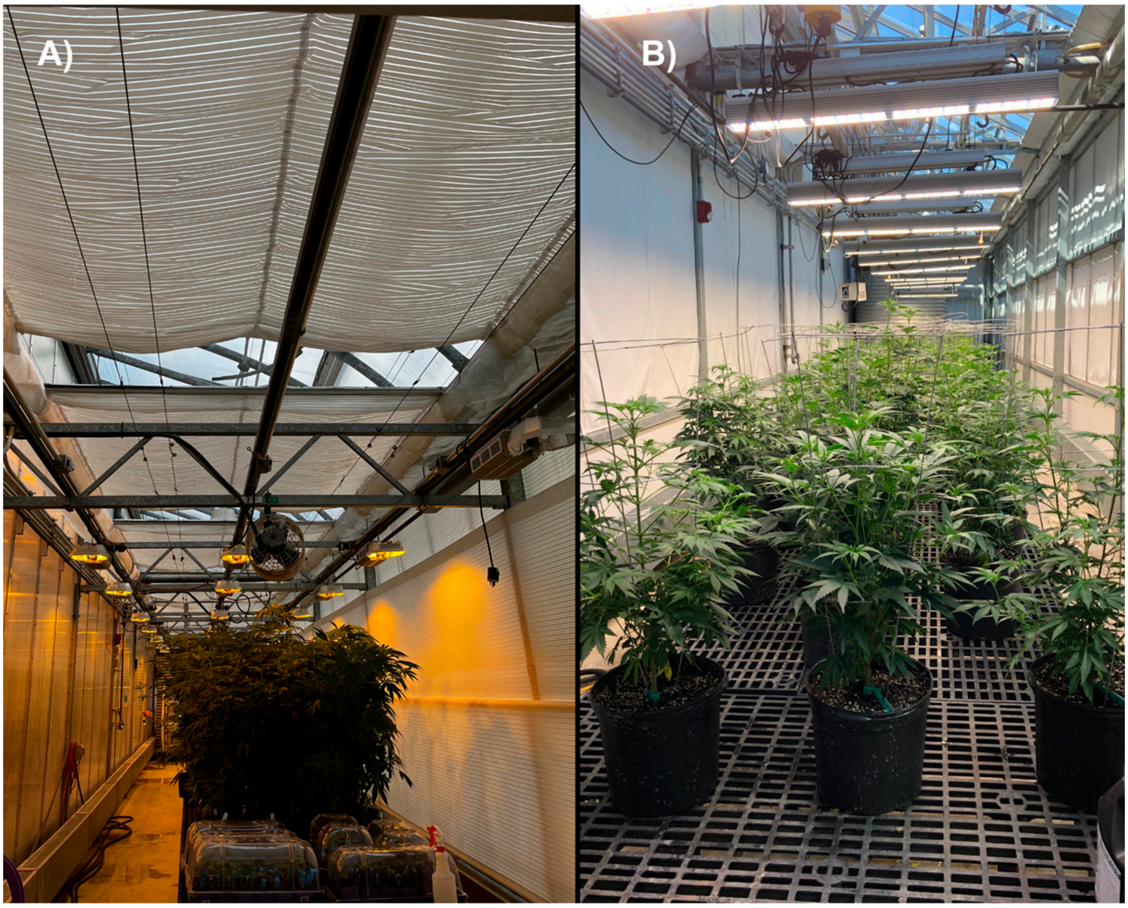

Supplementary Figure S1. Greenhouses compartments at Laval University, Quebec, Canada used for the phenotyping trials. (A) The vegetative compartment (B) The flowering compartment.

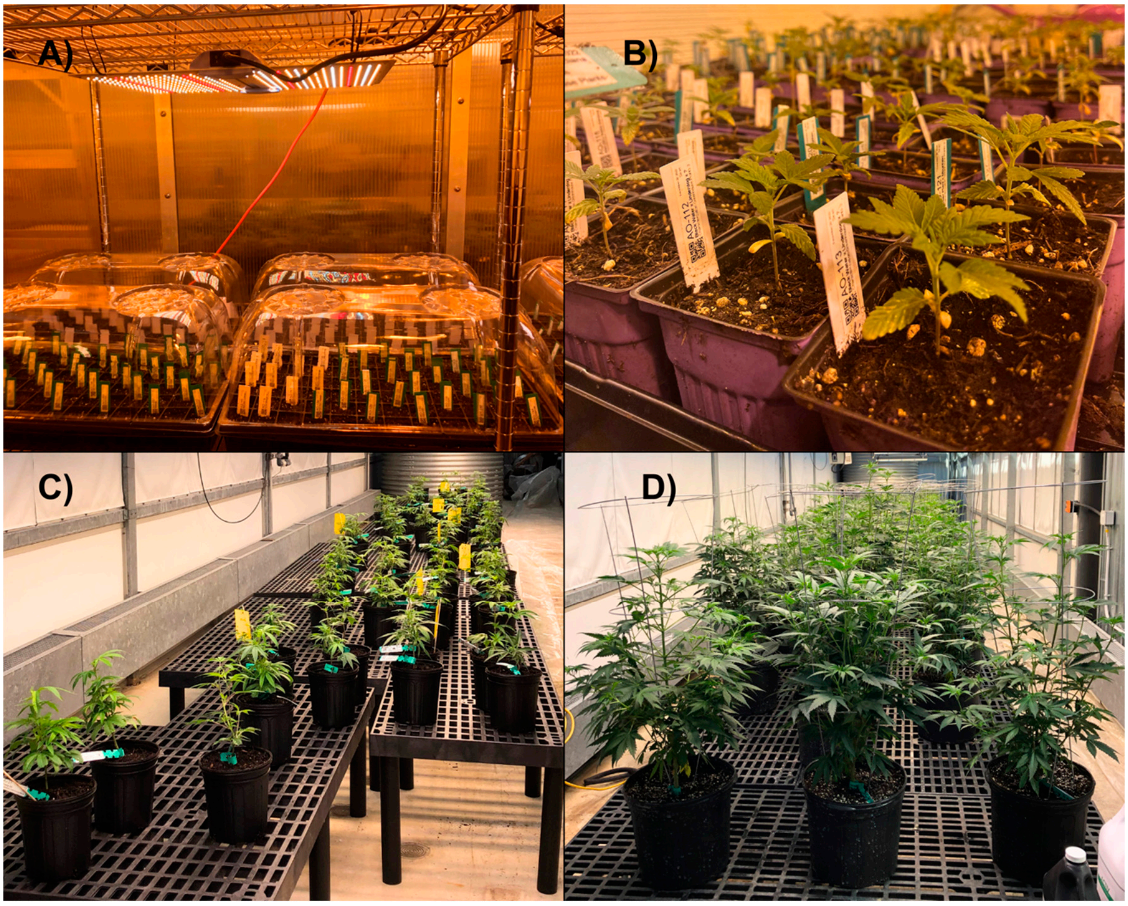

Supplementary Figure S2. Types of growth containers for plant development. (A) 72 multicellular trays, (B) 4-inches pot, (C) 1-gallon pots, (D) 5-gallons pots used from week 5 of vegetative through harvest.

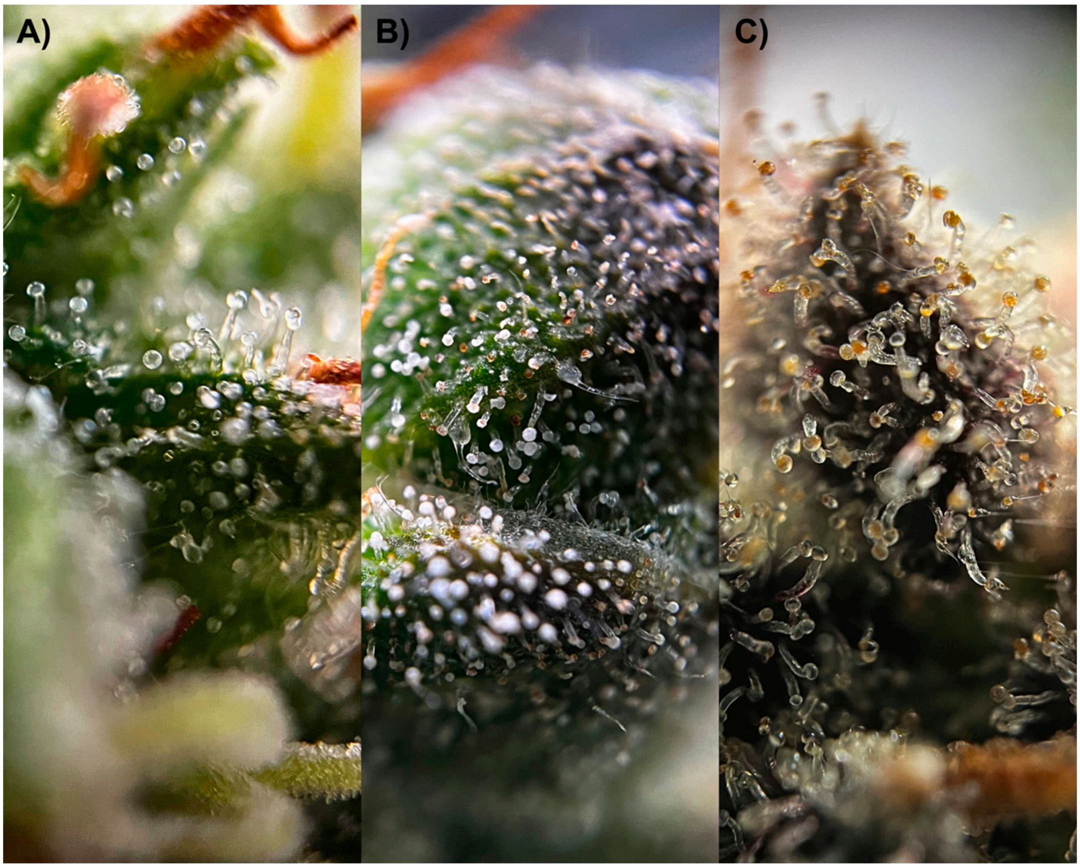

Supplementary Figure S3. The trichome coloration at different growth stages. (A) Displays clear trichomes, characterized by their transparent appearance. (B) Milky trichomes are depicted, displaying a cloudy and opaque coloration. (C) A mixture of amber and milky trichomes, representing a combination of golden hues and opaque shades.

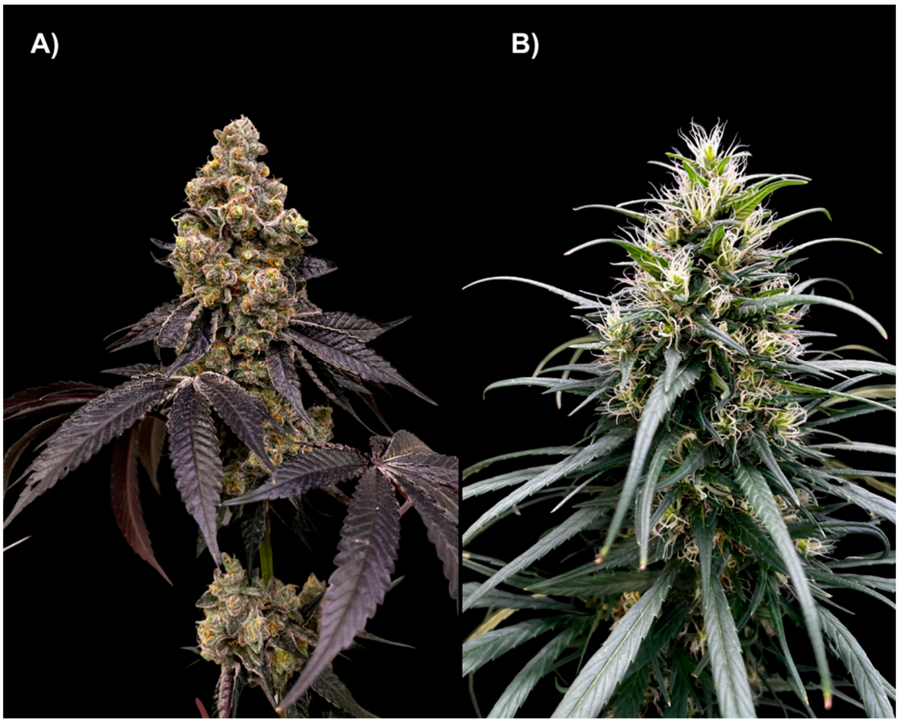

Supplementary Figure S4. A visual comparison of cannabis flower maturity. (A) A mature flower of cannabis plant cv. AN-14, displaying clear visual cues (e.g., color change of the leaf, darkening of the pistils, and compact flower shape) indicating maturity. (B) An immature flower of cannabis plant cv. AO-84, lacking evident visual indicators of maturity.

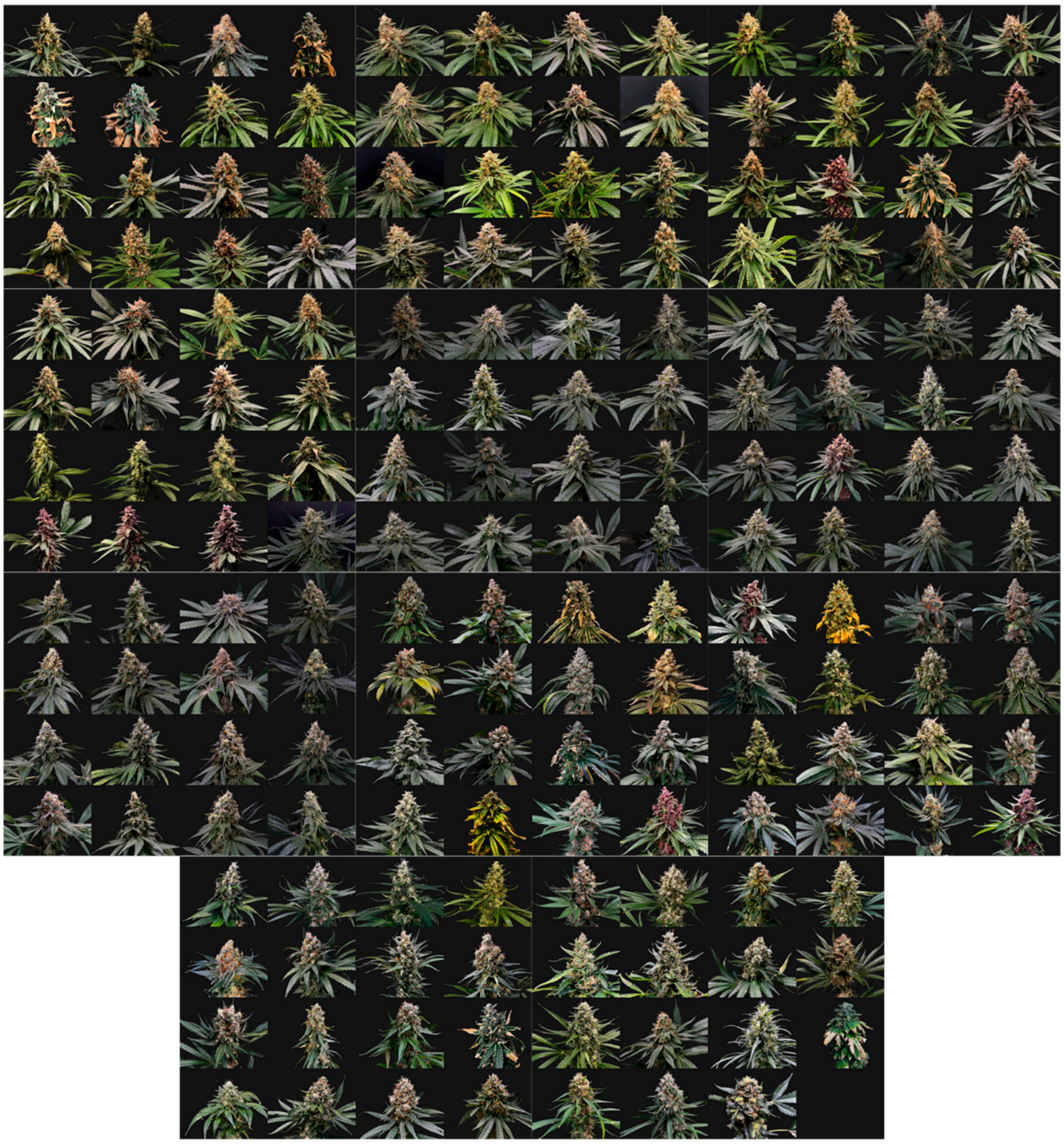

Supplementary Figure S5. Visual representation of the phenotypic diversity observed in the inflorescences of 175 out of the 176 accessions utilized in this study. We were unable to provide a photo for one accession (AJ-51). The figure is presented in a grid format with 11 blocks, each containing 16 photos. The order of photos in each blocks follows the same sequence as presented in Supplementary table 4: AG-3 to AJ-22, AJ-23 to AJ-37, AJ-38 to AJ-53, AJ-54 and AJ-55 and AN-

93 then AJ-56 to AJ-9, AN-10 to AN-14, AN-140 to AN-44, AN-45 to AN-91, AO-1 to AO-166, AO-168 to AO-214, AO-217 to AO-52, AO-56 to AO-98
